# Multilayer Casting of Eco-Efficient Self-Compacting Concrete with Reduced Binder Content

**DOI:** 10.3390/ma14195685

**Published:** 2021-09-29

**Authors:** Piotr Dybeł, Milena Kucharska

**Affiliations:** Faculty of Civil Engineering and Resource Management, AGH University of Science and Technology, 30-059 Cracow, Poland; kucharska@agh.edu.pl

**Keywords:** eco-efficient self-compacting concrete, multilayer casting, bond strength, placement technology

## Abstract

In the study, experiments were performed on two eco-efficient self-compacting concrete mixes of reduced binder content containing supplementary cementitious materials. The behaviour of the eco-efficient self-compacting concrete (SCC) mixture was examined to determine whether it is suitable for multilayer casting. It is recommended that the SCC should be poured in an uninterrupted manner. However, it is not uncommon that contractors are forced to take breaks as a result of delivery delays. Casting the elements in multiple poorly prepared layers may cause the creation of cold joints between them. Two technological variants of the multilayer casting of eco-efficient SCC on beam elements were analysed: pouring the mixture from a minor height on the previously placed layer and placing the subsequent layer on the mechanically disturbed surface of the underlying material. Different delay times were used: 15, 30, 45 and 60 min between the execution of two layers of eco-efficient SCC. The load-bearing capacity of the joint was determined using a splitting tensile strength test on cubic elements. It was observed that, regardless of the mixture and casting variant, the interlayer bond strength decreased as the delay time increased. This effect was less pronounced when the first layer was mechanically disturbed. It was also demonstrated that concrete with reduced binder content is characterized by a lower drop in bond strength between successive layers. Finally, it is noted that the current recommendations and normative guidelines for the multilayer casting of self-compacting concrete should be specified with regard to the time delay allowed for the execution of the next layer in the absence of interference with the previously placed layer. Lack of clarity in this respect may result in the creation of a cold joint and hence a reduction in the load-bearing capacity between layers.

## 1. Introduction

The largest contribution to the negative influence of concrete lies primarily in the significant environmental imprint of Portland cement and the substantial amount of concrete production overall. The related air and water pollution contributes to global warming as well as acidification and eutrophication [1]. In recent years, the possibilities of reducing its environmental impact have been investigated by developing new types of concrete, i.e., eco-efficient concrete, e.g., by introducing supplementary cementitious materials and recycled aggregates and wastes into mixtures. In the case of self-compacting concrete (SCC), which is already widely used on the market, it is recommended not only to replace cement with supplementary cementitious materials but also to reduce the amount of binder in general [2]. Furthermore, the positive outcome in terms of sustainability of SCC is enhanced by the elimination of additional mechanical compaction.

In the case of large-scale construction works such as high walls and considerable foundations, contractors often use multilayer casting, which involves the application of technological delays between successive layers of SCC. Such delays are usually caused by disruptions in the supply of the concrete mix. A higher viscosity layer can form on the surface of the already cast SCC mix, which, when not properly combined with new concrete, can create not only unaesthetic lift lines and discolorations, but most importantly reduce the bond strength of the joint [3,4,5], which may be further intensified by hot weather conditions. In the case of multilayer casting of normal concrete, the layer of concrete previously placed is excited by re-vibration [6,7], whereas in SCC the procedure of combining the layers is more complex due to its sensitivity to uncontrolled vibrations. In general, it is assumed that SCC shall be placed in a continuous and undisturbed manner [8]. However, in practice this is rare, and in accordance with some of the design guidelines [9,10], if a pause occurs, the surface should be carefully treated to regain flowability and proper mixing of the two layers, e.g., through surface mixing or vibrations. This can be achieved by either surface mixing or by using low vibration. In the case of pumping it is recommended that the concrete pressure in the pipeline should be increased. Alternatively, the free-fall distance of a new layer can be increased to obtain a greater impact on the pre-existing material. One should note that these procedures should be introduced with simultaneous control of segregation and bleeding, which must be prevented.

However, each standard specifies different methods that do not have a clear timeframe regarding when they should be used. The ACI standard [9] states that individual batches of the mixture should be available within a short time frame, while the SCC European Project Group recommendations [10] mention deciding on additional disturbance of the concrete layer if the live surface has crusted or stiffened to the extent that a cold joint or surface blemish could form. In addition, the recommendations do not take into account new types of self-compacting concrete such as reduced binder content concrete, i.e., eco-efficient SCC.

Given the guidelines for multilayer casting of SCC elements, an essential issue in self-compacting mixtures is their thixotropy. This time-dependent phenomenon is responsible for the continuous loss of apparent viscosity when there is shear and stiffening of the mixture during its rest [11,12]. The structural build-up at rest is considered to be a partially reversible process due to the effect of cement hydration. The extent to which the mixture returns to its original rheological properties has an impact on a number of technological factors, including pumpability, segregation, reduction in lateral pressure on the formwork, and bond strength between adjacent layers [13,14,15]. This phenomenon also would appear to be important for concretes with reduced binder content.

A considerable number of studies with new generation concretes [3,5,16,17] were conducted on the load-bearing capacity of the joint resulting from delays using different destructive tests. A reduction in mechanical strength of more than 40% was reported on the cylindrical core samples using a simple compression test [5]. Furthermore, in other studies on self-compacting mixtures with different levels of structural build-up at rest [3,4], for the delay time of 60 min, the residual bond strength varied in the range of 56–91% (slant shear test), 15–76% (direct shear test) and 56–80% (flexural strength test). Tests on three variants of fresh surface conditioning [16] indicated that the slightest reduction in bond strength between neighbouring layers occurring due to mechanical disturbance of prior layer and for a delay time of 45 and 60 min was 6% and 5%, respectively.

Given the fact that it is hardly ever possible to carry out continuous concrete placing works and there is increasing consumption of concrete of lower environmental impact, including eco-efficient SCC, the effect of introducing breaks into the concrete layer placing procedure ought to be analysed thoroughly in line with state-of-the-art placement technologies. Furthermore, the significant environmental footprint of concrete raises the issue of the implementation of eco-efficient concrete, which should also be examined in terms of multilayer casting. This study looks into the effect of multilayer casting technology on the load-bearing capacity of a joint between layers of eco-efficient SCC based on the testing of two different mixtures.

## 2. Materials and Methods

### 2.1. Concrete Mixtures

In the present study, an eco-efficient self-compacting concrete is assumed as a self-compacting mixture with a reduced total binder content. Two types of eco-efficient SCCs were used. One of them had a total binder content under 315 kg/m^3^ which, in the light of the ICI Rheocenter SCC classification [18,19], qualifies it as the Eco-SCC. Fly ash and silica fume were used as mineral additives, which additionally contribute to the positive environmental performance of the mix as these are waste materials. The second mixture with a total binder content of 385 kg/m^3^ ranks as Green-SCC [18,19] and was based on cement and fly ash. The specified densities of powders, based on product specification, were 3.1 g/cm^3^, 2.1 g/cm^3^ and 2.2 g/cm^3^, respectively, for cement, fly ash and silica fume. The mixtures were designed based on the literature [20] and adapted to the local resources and their parameters. The compositions of the mixtures are presented in Table 1. The water-to-binder ratio was assumed to be 0.5 and 0.45, respectively, for Eco-SCC and Green-SCC. Polycarboxylate with a polyethylene condensate defoamed based admixture played the role of a superplasticizer. The amount of superplasticiser was modified for each batch to achieve the desired flow and plastic viscosity of the mix and a slump-flow of 670 ± 10 mm. The normative guidelines classify the aggregates as G_C_85/20 and GF85 grading categories [21,22]. The bulk densities of the aggregates were 1.64 g/cm^3^, 1.62 g/cm^3^ and 1.53 g/cm^3^, respectively, for sand and gravel of fraction 2–8 mm and 8–16 mm based on [23].

### 2.2. Test Specimens

**Figure 1:**
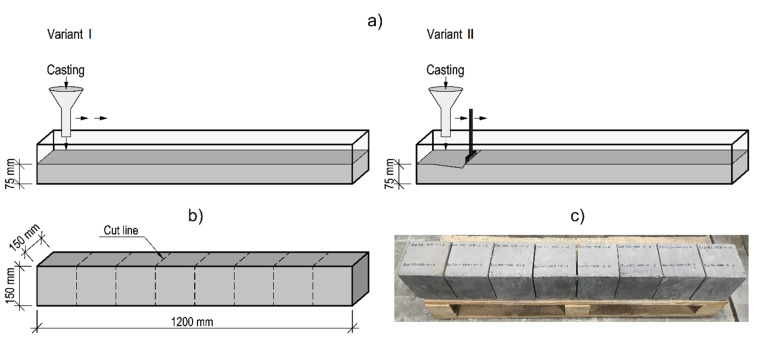
Specimens used: (**a**) a schematic view of surface preparation variants in multilayer casting; (**b**) a schematic view of a test element; (**c**) a test element cut into modules.

First, elements with dimensions of 1200 mm × 150 mm × 150 mm (length, height, width) were made (Figure 1b). The test element was designed to consist of 8 cubic base modules with dimensions of approximately 150 mm × 150 mm × 150 mm (Figure 1c). The execution of the specimens was performed in two phases. In the first phase, a layer of concrete was placed (from a single concrete batch) up to a half of the element height, i.e., 75 mm. In the second stage, the moulds were filled up to a height of 150 mm using the next batch of the same eco-efficient SCC. The simulation of a time interruption in placing the mix was achieved by delaying the execution of the second phase. The pause between placing a subsequent layer of Eco-SCC mix was defined as 15, 30, 45 and 60 min, and as 30 and 60 min in the case of Green-SCC mix. The fresh eco-efficient SCC mix used in the second stage was stored in a concrete mixer until casting. For each time frame, two different technologies of placing the mix and preparation of a first layer surface were used, the schematic views of which are shown in Figure 1a. Variant I assumed no interference with the first layer of mix. It was performed by placing the mix from the container located about 5 cm higher than the surface of the already placed layer. Variant II included mechanical disturbance of previously laid layer with a trowel to the depth of approx. 3 cm in order to restore its former plastic state and subsequent placement of another layer of the mix from a height of 5 cm. In both variants, the mixture was distributed all over the element, without adopting one fixed casting point.

After 3 days of concrete curing, the formwork was stripped. The elements were stored in a fixed position in the laboratory under a temperature of 20 °C and relative humidity of 95% throughout the concrete ageing period. Twenty one days later, the elements were chopped into elementary modules and the experiments were conducted after 28 days. Eight samples were used in each test series. Thus, a total of 96 base modules were prepared. In addition, 10 reference samples cast in a single layer were extracted from each batch and dedicated to tests of compressive and splitting tensile strengths.

### 2.3. Test Procedures

#### 2.3.1. Fresh and Hardened Properties of Concrete

Fresh eco-efficient self-compacting concrete compositions were subjected to three tests of their flow properties. Both flowability and fluidity were identified on the basis of slump-flow test [24]. The following flow parameters were measured: the final slump–flow diameter and the time T_500_—time corresponding to a diameter of 500 mm. The L-box test was used to evaluate the passing ability of the eco-efficient SCCs [25]. Furthermore, the Fresh Visual Segregation Index was assessed after performing the slump–flow test and allowed on determining the segregation resistance [26]. The flow tests of the first batches (Eco-SCC I and Green-SCC I) were carried out as soon as the mixing was finished (t = 0 min). In the case of Eco-SCC II, the flow tests were performed just before the end of mould filling and 15, 30 and 45 min after mixing finish (t = 0 min, t = 15 min, t = 30 min, t = 45 min, respectively). In the case of Green-SCC II, the test were conducted two times: before the casting (t = 0 min) and 30 min after mixing finished (t = 30 min)

From each batch of concrete, 5 reference samples were prepared to perform the compressive strength test according to EN 12390-3 [27]. In addition, 5 specimens of each batch were prepared for determining the splitting tensile strength according to EN 12390-6 [28]. The tests were carried out on cubic 150 mm × 150 mm × 150 mm samples 28 days after curing in standard laboratory conditions (temperature 20 °C, relative humidity 95%).

#### 2.3.2. Test of the Load-Bearing Capacity of a Joint

**Figure 2:**
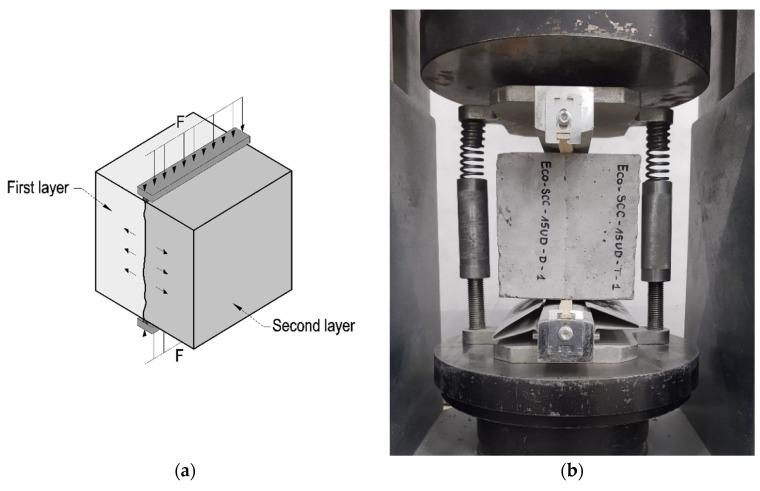
A view of: (**a**) sketch of test setup; (**b**) test of bond strength on typical sample.

The load-bearing capacity of a joint between two layers of eco-efficient SCCs was investigated using 150 mm × 150 mm × 150 mm samples under splitting tension following the standard procedure [28]. The experiments were conducted 4 weeks after maturing in lab conditions. In each case, the load was applied at the surface–surface contact zone of the specimen formed by the formwork. This guaranteed mutually perpendicular surfaces and adequate smoothness. The interlayer zone was oriented vertically along the axis of the forces applied in the case of both mix types. The schematic and real view of the test setup are given in Figure 2.

The load-bearing capacity of the joint was computed according to the procedure used to specify the concrete splitting tensile strength using the formula presented in Equation (1):

(1) $$f_{\mathrm{ct},\mathrm{sp}}=\frac{2F}{\pi \mathrm{Ld}}$$

where: f_ct,sp_—splitting tensile strength, [MPa]; F—peak load, [N]; L—the length of the sample contact line, [mm]; d—declared cross-sectional dimension, [mm].

## 3. Results

### 3.1. Fresh and Hardened Properties of Concrete

The results of the fresh mix properties are presented in Table 2. Based on the results, one could tentatively classify both batches of Eco-SCC and Green-SCC into the same slump–flow class (SF2) and the same passing ability class (PL2) in the nearly whole scope of the study. The assumption of approximately 670 ± 10 mm slump–flow was achieved. The preceding statements do not apply only to the case of the Green-SCC batch after 30 min of delay time, as its slump-flow diameter was 655 mm (SF1 class). As for fluidity, the delay time influenced the slump-flow time T_500_, thus classifying the mixtures to both VS1 and VS2 classes, depending on the rest time. In the case of the second batch, in order to maintain similar flow properties, an additional amount of superplasticizer was added during resting time. Moreover, the eco-efficient SCC mixtures stayed stable and showed no signs of segregation nor bleeding during the testing time. Comparing both types of eco-efficient concrete, the Green-SCC was characterized by a higher viscosity and lower slump–flow than Eco-SCC, which resulted in a lower L-box ratio that still was in a normative passing ability range. The L-box ratio could also be affected by a contribution of coarse aggregate in a mixture; as Green-SCC had a higher volume of coarse aggregate than Eco-SCC, the L-box ratio was expected to be lower for Green-SCC [29].

The results of the compressive strength tests performed on the reference samples (cast in a single layer) with their standard deviation (SD) are demonstrated in Table 3. The samples were cast immediately after the completion of fresh properties tests. Given the proper laboratory conditions while mixing and correct proportions of constituent materials, the results of both batches did not differ significantly. The difference between SCC batches were a result of the scatter, yet the strength parameters remained similar. A slightly higher compressive strength of Green-SCC was a result of higher cement content and thus lower water-to-binder in the mixture.

### 3.2. The Load-Bearing Capacity of a Joint between Two Layers of Eco-Efficient SCC

Table 4 summarises the results of the tests of splitting tensile strength performed on cube modules derived from beam elements and their coefficients of variation. The values of splitting tensile strength for samples with no delay time were adopted as a mean of the results obtained on 5 cubic reference samples of each batch, that is 10 samples for Eco-SCC and 10 samples for Green-SCC. The splitting tensile strength of Green-SCC is greater than Eco-SCC which is related to its higher compressive strength. It should be noted that in the case of both variants of surface preparation, the duration of the delay between successive layers had a negative effect on the load-bearing capacity of a joint between both Eco-SCC and Green-SCC layers. A greater drop of splitting tensile strength was found in Variant I. The reduction was approximately 24.3% in the case of 60 min delay time in relation to the reference sample. When it comes to Variant II, i.e., the disturbance of the surface structure, the decrease after 60 min of delay time equated to 10.5%. The slightest reduction in load-bearing capacity was noticed for the samples with a 15 and 30 min delay time in Variant II. As for Green-SCC, it was noticed that reduction in splitting tensile strength was bigger than in Eco-SCC. After 60 min of delay time, the decrease in surface preparation was approximately 28.7% and 6.7% for, respectively, Variant I and Variant II. Therefore, the mechanical disturbance of the first surface noticeably improves the quality of the bond between the Eco-SCC and Green-SCC layers.

The parameter describing the dynamics of a decrease in load-bearing capacity of a joint with respect to one minute is also presented in Table 4. The comparison of the results indicated that the reduction rate for Variant I was over two times higher than for Variant II in Eco-SCC and over 3 times higher in the case of Green-SCC. The largest reduction rate in time was observed in Green-SCC with Variant I of surface preparation. Yet, the values of the reduction rate in the whole range of tests were less than 0.01 MPa/min.

### 3.3. Effect of Surface Preparation on the Load-Bearing Capacity

**Figure 3:**
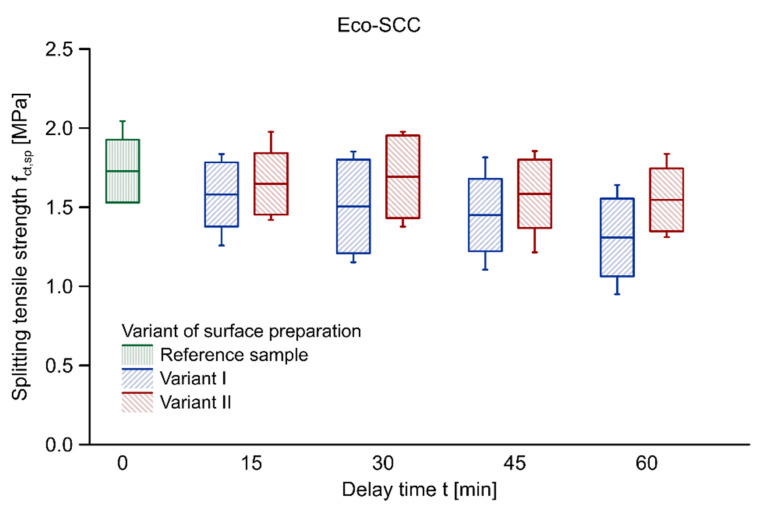
Splitting tensile strength vs. delay time between layers of Eco-SCC concrete.

**Figure 4:**
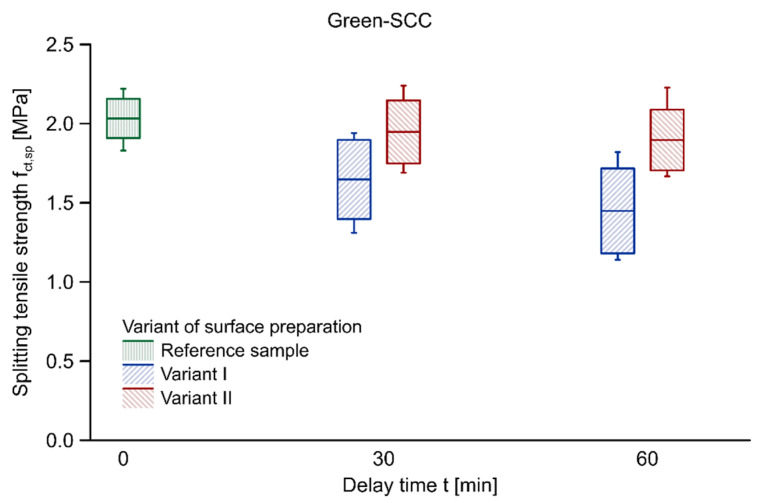
Splitting tensile strength vs. delay time between layers of Green-SCC concrete.

Figure 3 and Figure 4 illustrate the ranges of splitting tensile strength values using box plots with regard to samples made with Eco-SCC and Green-SCC, respectively. The diagrams show results with respect to the average value of splitting tensile strength. The boxes represent the standard deviation in each case, and the whiskers indicate the minimum and maximum values. The results obtained for both variants exhibited a big scatter; however, it was more significant in the case of Variant I in both types of concrete. The high value of scatter of this surface preparation technology, especially in the cases of 30 min delay time in Eco-SCC and 60 min delay time in Green-SCC, resulted from, among other things, the substantially lower splitting tensile strength of the outer samples in the test elements. The first and the last base modules suffered from the smallest area of proper layer–layer mixing, which resulted from the placing technology of the second layer.

**Figure 5:**
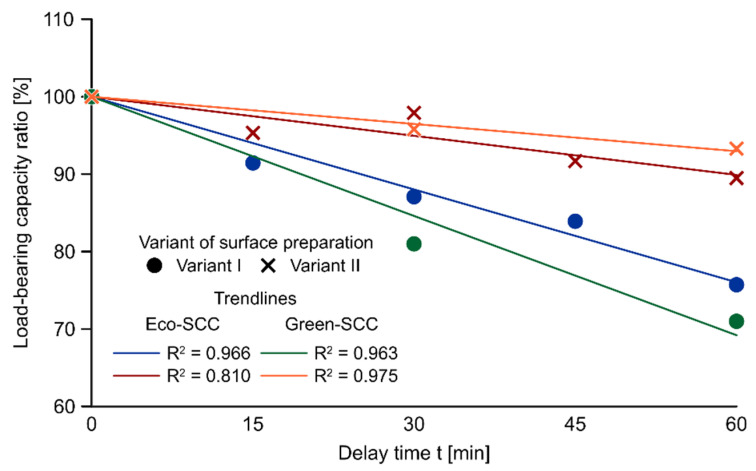
The relation between the load-bearing capacity ratio and delay time of subsequent layers.

The relation between the average load-bearing capacity ratio and the interval time for each variant of surface preparation, with the corresponding linear trendlines, are presented in Figure 5. The load-bearing capacity ratio was taken as the comparison of the mean of the delay times measured for the samples and the reference system. Regardless of the surface preparation, a decreasing tendency with a growing delay time between successive layers of Eco-SCC was observed for the whole scope of the study. A more significant reduction and strong linear dependence (R^2^ = 0.966) with a clear negative slope were noted for Variant I. In the case of Variant II, the drop was relatively minor and the coefficient of determination (R^2^ = 0.81) did not indicate a clear linear relationship. The same relationship was observed for elements made with layers of Green-SCC. In the case of both variants of placement technology, the tendency of the reduction in load-bearing capacity with the delay time increase was noted. A more pronounced effect of surface preparation was observed for the more viscous Green-SCC. Comparing the load-bearing capacity ratios for 60 min delay time in both types of eco-efficient SCC, the difference between the values found for Variant II and Variant I was approximately equivalent to 14% and 22% for Eco-SCC and Green-SCC, respectively.

### 3.4. Failure Mechanism of the Joint between Two Layers of Eco-Efficient SCC

**Figure 6:**
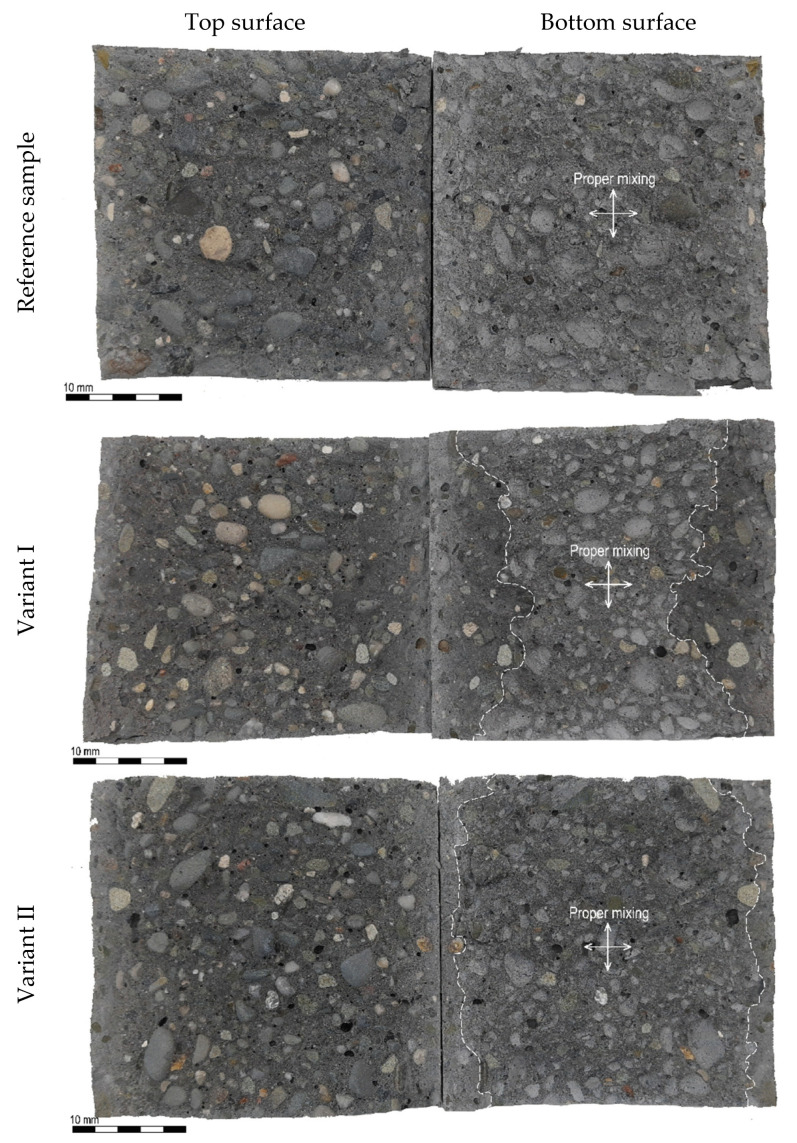
The examples of Eco-SCC specimen surfaces after splitting tensile strength test.

In an attempt to explain the phenomena responsible for the decrease in the load-bearing capacity of the joint between two eco-efficient SCC layers, the failure surface condition after splitting tensile strength tests was analysed. In both types of eco-efficient SCC, the failure mechanisms of each variant of surface preparation were alike. The typical failure surface condition after splitting tensile strength investigation for the reference specimen and two variants of surface preparation for a delay time of 30 min between layers of Eco-SCC is presented in Figure 6. The load-bearing capacity of the joint at the interface between successively placed layers of eco-efficient SCC mixes was determined by appropriate mixing of the two layers. In order to identify the failure mechanism of the successively placed layers as well as their level of proper mixing for each variant of surface preparation, the surfaces of the specimens after splitting tensile strength tests were analysed, using the close-shot photos.

In the case of the reference samples, the failure plane passed through the contact zone of the binder matrix and the aggregate. This is a commonly observed failure mechanism for concretes with low and medium strength properties. This results from a different microstructure of the hydrated matrix in the direct proximity of the aggregate grains as compared to the other areas. In the vicinity of the aggregate grains, the matrix is generally more diluted and thus more porous. Microcracks in the matrix–aggregate interface are therefore the primary reason for the failure of the bond strength [30].

In Variant I of surface preparation, the zone of sufficient layer mixing was situated in the middle part of the specimen cross-section where the fresh mix had impacted the incompletely solidified layer from a low height. In this area, the second layer mix penetrated the first layer forming a high-quality joint. The observed failure plane of the joint was similar to the reference samples. The dimension of the proper mixing zone depended on the delay time. The longer the time between laying the first layer and the subsequent one, the smaller the zone of proper mixing was. In the other parts of the sample, the second layer moved across freely over the first layer that had partially stiffened. The shear stress was not sufficient to reverse the process of structural build-up at rest. As a result, there was no mixing of the first and second layers. The failure in these zones was mainly caused by the debonding of the interlayer joint as it passed along the surface of the structural build-up. Hence, the joint was structurally comparable to a joint between hardened and new concrete. The research revealed that the larger the area of the undisturbed surface of the mix laid earlier, the lower the load-bearing capacity of a joint between two layers (Table 4, Figure 5).

In the case of Variant II of the surface preparation, the occurrence of the proper mixing of both layers was observed and the failure mechanism was similar to the reference samples made without interruption in concreting. Only at the edges of the specimens were there minor areas of failure between the second layer and the first layer, where structural build-up was noted. Variant II of the surface preparation caused adequate plasticisation of the previously laid mixture, thus facilitating good mixing with a fresh mixture.

## 4. Discussion

The results of the research allowed us to propose guidelines for casting eco-efficient SCC in layers so as to limit the effect of delays between layers on the mechanical strength of the beamlike structures, which assume the process of restoring a plastic state of the mixture that was previously made by mechanical surface disturbance (Variant II of placement technology). In this variant, no significant reduction in the load-bearing capacity of a joint between two layers of both Eco-SCC and Green-SCC was noted in the analysed delay time range. The effective performance of Variant II was a result of a uniform and limited interference of the mechanical disturbance across the whole joint surface. Analysis of the failure mechanism of specimens made in this variant showed a similar mechanism to monolithic samples. Moreover, in the case of this method, it was possible to eliminate traces and discolorations on the concrete surface (lift lines) at the interface between layers. However, it ought to be kept in mind that this sort of interference may trigger unwanted segregation, hence it should be performed with a great amount of control and following previous schedules.

Variant I of placement technology resulted in a greater decrease in the load-bearing capacity of a joint between two layers of eco-efficient SCCs. The inspection of the failure surface condition revealed that in this variant the satisfactory mixing of successive layers is limited as a result of a partial structural build-up in the rest of the first layer mixture and insufficient impact-induced plasticisation. As the intact surface area of the of the base layer increases, the load-bearing capacity of a joint between the two layers decreases. For this variant, a clear linear dependence of the decrease in load-bearing capacity of a joint with delay time was recorded in both types of eco-efficient SCC. Similar observations were reported by Megid and Khayat [3,4], that regardless of the composition and properties of the SCC mixes, a decrease in the bond strength of adjacent layers was noticed with the increase in the duration of delay time.

The authors drew analogous conclusions in their previous publication based on self-compacting concrete with a binder content of 450 kg/m^3^ [16]. That mixture exhibited stronger thixotropic behaviour and the variant of casting the mixture onto an undisturbed surface resulted in greater deterioration of the load-bearing capacity of the joint between successive layers. For a delay time of 60 min, the reduction in bond strength was 36% for this scenario. The decrease in this parameter was also in a linear relationship with delay time, as in Megid and Khayat [3,4]. Moreover, the variant of a mechanical disturbance of the surface was applied there, for which the reduction in bond strength was 5% for 60 min delay time. In comparison with the Eco-SCC findings, which are 24.3% and 10.5%, respectively, it could be assumed that the interference with the surface structure is more efficient for more thixotropic mixtures, which further corresponds to the results of Green-SCC, as the reduction in bond strength in the 60 min delay time case was 29% and 6.5%, respectively, for Variant I and II. It is crucial to note that the thixotropic behaviour is strongly dependent on the amount of powder and the water-to-powder ratio [5]; hence, Eco-SCCs with considerably reduced binder content will be more convenient in the multilayer casting technology than traditional SCCs. It should be noted that less thixotropic mixes will exert a higher formwork pressure.

Furthermore, the failure mechanism under splitting tensile in eco-efficient SCCs was found to be more comparable to the behaviour of normal concrete rather than self-compacting concrete. The reason for this is the low powder content in the mixture and the more porous cement matrix in the aggregate area, which consequently cause failure along the interface between the matrix and the aggregate.

It should be taken into account that the occurrence of a partially reversible structural build-up at rest may be of importance for the layer-to-layer bond strength when placing concretes according to the international guidelines [9,10,31] also in the cases of concretes with reduced binder content. In general, the guidelines instruct manufacturers to place concrete from an individual casting point positioned at one mould edge up to the formwork being completely filled for all self-compacting mixtures. It is paramount to ensure a mixture flow over a certain distance to secure self-venting and self-levelling. This method is applicable in the case of small elements and/or continuous mix flow. If the concrete work is interrupted, the placing of successive layers from a single casting point could be inappropriate and result in weaker joints at the interface of subsequent layers, when flowing over the partially stiffened previous layer. Consequently, this could result in reduced strength characteristics and durability of the hardened concrete.

## 5. Conclusions

From the experimental studies conducted, a set of conclusions and recommendations can be drawn:

- In general, a decrease in the load-bearing capacity of a joint between two layers of eco-efficient SCC was observed with an increase in the duration of the delay time. The most substantial reduction was found for the technological variant connected with the free-flowing of the mixture over the previously laid layer. For delay times of 15, 30, 45, 60 min in the case of Eco-SCC, the average decrease in bond in relation to the reference sample was 8.5%, 12.9%, 16.1% and 24.3%, respectively. In the case of Green-SCC, for which delay times of 30 and 60 min were investigated, the reduction in relation to reference samples were 18.9% and 28.7%, respectively. In the technology variant connected with mechanical disturbance of the previously placed layer, a slight decrease, ranging between 2.1% and 10.5%, was obtained for Eco-SCC, and between 4.2% and 6.7% for Green-SCC.
- The current recommendations and normative guidelines for multilayer casting of self-compacting concrete appear to be insufficiently detailed regarding the time of delay between layers if there is no interference with the previously placed batch. On the basis of this research, in the case of concreting with intervals longer than 15 min between layers, it is recommended that a fresh layer should follow superficial plasticisation of the underlying layer to improve the bond at their interface.
- Due to its reduced binder content, eco-efficient SCC concrete is characterised by reduced structural build-up at rest, which contributes to a smaller decrease in the load-bearing capacity of a joint between two layers compared to traditional SCC concrete. In general, eco-efficient SCC would be more convenient in the multilayer casting technology than traditional SCCs that are rich in powder content.

## Figures and Tables

**Table 1:** Proportioning of investigated eco-efficient SCC mixtures for 1 cubic meter.

Constituent Materials	Compositions	
	Eco-SCC [kg/m^3^]	Green-SCC [kg/m^3^]
Cement CEM I 42.5R	218	285
Water	152	174
Sand 0–2 mm	922	700
Gravel aggregate 2–8 mm	540	530
Gravel aggregate 8–16 mm	367	530
Fly ash	75	100
Silica fume	11	-
Superplasticizer	2.61	2.44

**Table 2:** Fresh properties of eco-efficient SCC mix batches.

Mix Batch	Delay Time [min]	Slump-Flow [mm]	Slump-Flow Class	Slump-Flow Time T_500_ [s]	Viscosity Class	Fresh Visual Stability Index	L-Box Ratio	L-Box Class
Eco-SCC I	t = 0 min	670	SF2	1.6	VS1	0	0.89	PL2
Eco-SCC II	t = 0 min	680	SF2	1.8	VS1	0	0.92	PL2
Eco-SCC II	t = 15 min	665	SF2	2.0	VS2	0	0.87	PL2
Eco-SCC II	t = 30 min	675	SF2	1.8	VS1	0	0.89	PL2
Eco-SCC II	t = 45 min	670	SF2	1.7	VS1	0	0.90	PL2
Green-SCC I	t = 0 min	660	SF2	2.2	VS2	0	0.85	PL2
Green-SCC II	t = 0 min	660	SF2	2.3	VS2	0	0.89	PL2
Green-SCC II	t = 30 min	655	SF1	2.5	VS2	0	0.84	PL2

**Table 3:** Results of compressive strength tests.

Mix Batch	Compressive Strength	
	f_cc_ [MPa]	SD [MPa]
Eco-SCC I	28.06	0.57
Eco-SCC II	30.05	2.10
Green-SCC I	33.40	1.67
Green-SCC II	34.16	2.73

**Table 4:** Results of splitting tensile strength measurements on samples made of multiple concrete layers.

Concrete Mix	Variant of Surface Preparation	Delay Time between Successive Layers										Reduction Rate in Load-Bearing Capacity [MPa/min]
		t = 0 min		t = 15 min		t = 30 min		t = 45 min		t = 60 min		
		f_ct,sp_ [MPa]	SD [MPa]	f_ct,sp_ [MPa]	SD [MPa]	f_ct,sp_ [MPa]	SD [MPa]	f_ct,sp_ [MPa]	SD [MPa]	f_ct,sp_ [MPa]	SD [MPa]	
Eco-SCC	Variant I	1.73	0.19	1.58	0.21	1.50	0.30	1.45	0.23	1.31	0.25	0.00699
	Variant II			1.65	0.20	1.69	0.25	1.58	0.22	1.55	0.20	0.00303
Green-SCC	Variant I	2.03	0.12	-	-	1.65	0.25	-	-	1.45	0.26	0.00973
	Variant II			-	-	1.95	0.20	-	-	1.90	0.19	0.00227

## Data Availability

The data that support the findings of this study are available from the corresponding author upon request.
